# Severe acrocyanosis precipitated by cold agglutinin secondary to infection with *Mycoplasma pneumoniae* in a pediatric patient

**DOI:** 10.3325/cmj.2017.58.424

**Published:** 2017-12

**Authors:** Bernadett Mosdósi, Zoltán Nyul, Arnold Nagy, Kata Bölcskei, Tamás Decsi, Zsuzsanna Helyes

**Affiliations:** 1 Department of Pediatrics, Clinical Center, University of Pecs, Pecs, Hungary; 2 Department of Pharmacology and Pharmacotherapy, Medical School & János Szentágothai Research Center for Neuroscience, University of Pécs, Pécs, Hungary; 3 MTA-PTE NAP B Chronic Pain Research Group

## Abstract

This is the first report describing a severe form of cold agglutinin-induced acrocyanosis with cutaneous necrosis after Mycoplasma infection in a 9-year-old patient without any other severe symptoms and laboratory alterations. We also present the results of two non-invasive methods used to determine the viability of tissues, degree of tissue perfusion impairment, and the responsiveness of the microvasculature. Laser Doppler flowmetry and laser speckle contrast imaging, both suitable to measure tissue blood perfusion non-invasively, have been used in the diagnosis and follow-up of various peripheral vascular diseases. In our patient, we demonstrated remarkably reduced microcirculation before the treatment and a significant perfusion increase in the acral regions after pentoxifylline therapy. The investigational techniques were useful tools to assess and quantify the severity of peripheral perfusion disturbances and to monitor the efficacy of the treatment in our patient.

Acrocyanosis is a vascular disease usually characterized by persistent, non-paroxysmal, bluish-red symmetrical discoloration of the hands and feet. It may also affect the ear lobes and the tip of the nose. The disease usually manifests before the age of 25. Acrocyanosis can occur in the primary form without detectable previous disease or in the secondary form resulting from specific disorders, such as cold agglutinin syndrome (CAS) (1-3).

Together with paroxysmal cold hemoglobinuria (PCH), CAS is classified into the group of cold autoimmune hemolytic anemias, representing a subgroup of autoimmune hemolytic anemias. The diagnosis of these clinically distinctive disorders is based on their characteristic serologic reactions. CAS is etiologically further divided into idiopathic and secondary forms caused by underlying non-malignant or malignant diseases (3).

In contrast to PCH, CAS is generally caused by IgM autoantibodies that exhibit their maximum reactivity at 4°C. Cold agglutinins (CA) circulate in nearly everyone, but in low titer (3,4), whereas clinically significant agglutinins occur at titers of 1:1000 with thermal activity range extended toward warmer temperatures. When blood temperature drops below the thermal maximum of the antibody, IgM binds to erythrocytes and causes agglutination and complement activation responsible for hemolysis. The clumped erythrocytes may occlude peripheral microvasculature, which may lead to acrocyanosis and, in severe cases, ischemic gangrenes.

The characteristic clinical manifestations of CAS consist of hemoglobinuria, acrocyanosis, Raynaud’s phenomenon, cutaneous necrosis, and occasionally gangrene. The symptoms appear or worsen typically at cold temperature (2,3,5), but the extent of the hypoperfusion is not routinely measured in the clinical practice.

To the best of our knowledge, this is the first case report of severe acrocyanosis precipitated by cold agglutinin secondary to infection with *Mycoplasma pneumoniae* in a pediatric patient. A further novelty of the case is the use of two non-invasive techniques, not used in routine practice, to determine the severity of microcirculatory impairment and evaluate the effectiveness of therapy.

## CASE REPORT

A 9-year-old Caucasian girl, born at 39 weeks of gestation after an uneventful pregnancy, had been taking inhaled steroid (fluticasone proprionate) medication for asthma for 2 years, but otherwise had an unremarkable medical history. She had undergone all routine immunizations at the appropriate ages.

The patient had become subfebrile and developed unproductive cough two weeks before admission to hospital. She was treated with amoxicillin-clavulanate antibiotic and fluticasone proprionate and salbutamol inhalation. One week later, painful, bluish discoloration of the fingers occurred, which was worsening in cold temperature. She was referred to our emergency unit.

**Figure 1:**
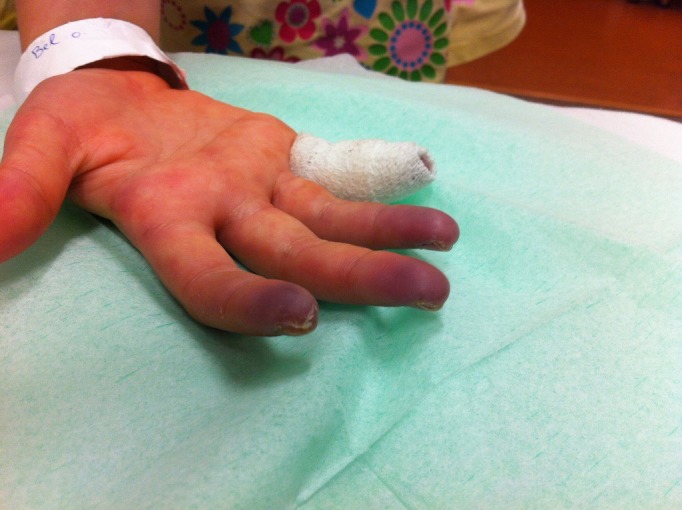
Severe acrocyanosis with cutaneous necrosis in the 9-year-old female patient (photo taken at the time of first admission).

On physical examination, the patient’s fingers and toes were cold, blue, and tender, with a small cutaneous necrosis of the digital phalanx of the fourth finger (Figure 1). Sclerodactyly was not observed. Radial and axillary pulses on both sides were equal and regular, and blood pressure and heart rate were in the normal range. The elevated arm test was not indicative for thoracic outlet syndrome. Crepitation was observed above the middle lobe of the right lung. No further abnormalities were seen on physical and neurological examination.

Routine laboratory tests including hematocrit (Htc), hemoglobin (Hgb), white blood cell count (WBC), platelets, differential blood smear, C- reactive protein (CRP), lactate dehydrogenase (LDH), transaminases, thyroid stimulating hormone (TSH), coagulation parameters, and serum creatinine. All laboratory findings were in reference ranges, while erythrocyte sedimentation rate (ESR) was slightly increased. Urinalysis was not indicative for hemolysis. Immunoglobulin and complement C3 and C4 levels were also normal and the screening test excluded the presence of cryoglobulins. Direct antiglobulin test (Coombs test) detected the presence of both IgM and C3d. IgM exhibited a peak auto-agglutination at 4°C; its reactivity gradually vanished above room temperature and became inactive at 37°C (Table 1).

**Table 1:** Thermal activity of cold agglutinins at different time points

	Cold agglutinins activity*			
Date of examination	4°C	15°C	20°C	25°C
December 10, 2014	++++	++	+	-
May 20, 2015	+++	++	+/−	-
January 10, 2016	+++	++	-	-

*The positive agglutination reaction is graded by a semiquantitative score from + to 4+.

Immunoserological investigations revealed increased anti-nucleosoma antibody (76.9 UI/mL, normal <20 UI/mL), while other autoantibodies (anti-nuclear, anti-dsDNA, anti-centromere, anti-C1Q, extractable nuclear antibody, anti-saccharomyces cerevisieae antibodies, anti-neutrophil cytoplasmic antibodies, anticardiolipin and anti-beta 2 glycoprotein, and antiprothrombin) were in the normal range. Infectious serology results for Epstein-Barr virus, cytomegalovirus, Varicella zoster virus, human immunodeficiency virus (HIV)-1,-2, Parvovirus B19, Hepatitis -A, -B, -C, -E virus, Chlamydia pneumoniae excluded recent infections. The antistreptolysin-O titer was normal. The venereal diseases research laboratory test VDRL was non-reactive. Mycoplasma IgM and IgA antibodies were markedly increased, whereas IgG was slightly increased, ie, these findings indicated recent *Mycoplasma pneumoniae* infection.

Chest x-ray revealed pneumonia in the right lobe. Abdominal ultrasound findings were normal. Echocardiography showed normal cardiac anatomy, and the patient had good peripheral pulses with normal flow on Doppler examination. Ophthalmological examination was not indicative for vasculitis. Ramified capillaries were observed with capillary microscopy.

On the basis of clinical examination and laboratory test results, we reasonably suspected that the girl had acrocyanosis precipitated by cold agglutinin secondary to *Mycoplasma pneumoniae* infection. Autoimmune disorders and drug-induced acrocyanosis were ruled out on the basis of medical history and laboratory test results.

The patient was treated for 2 weeks with oral clarithromycin and intravenous pentoxyphyline infusions (200 mg/dose) three times a week in the beginning. No adverse events occurred during pentoxyphyline treatment. After the first 3 months of therapy, the frequency of infusions was set to every 2-3 weeks according to the improving clinical signs and continued for 12 months every three weeks. No plasma exchange therapy was needed. The patient was advised to avoid cold temperatures and keep her peripheries warm, eg, by wearing gloves. There were no problems with adherence to medical advice.

Since the symptoms persisted for over two months after the first admission, we decided to use two non-invasive methods to determine the viability of tissues, the degree of tissue perfusion impairment, and the responsiveness of the microvasculature.

**Figure 2:**
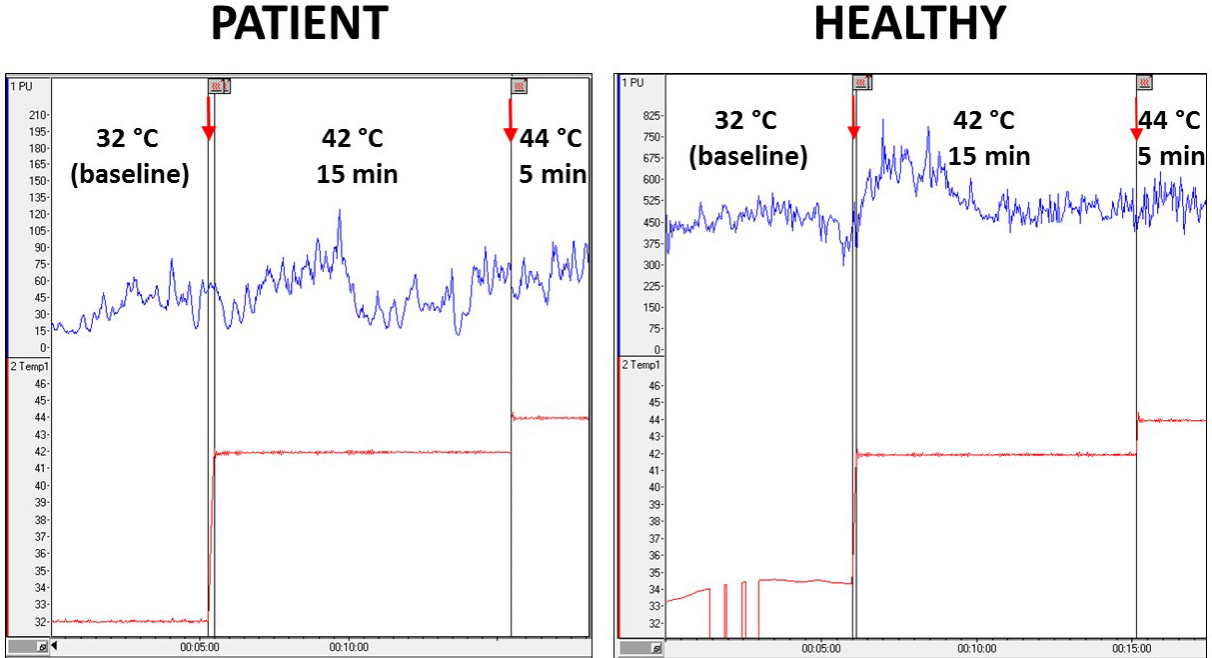
Measurement of blood flow with the Periflux 5000 system at basal temperature (I., at 32°C, 5 minutes), and after heating to 42°C (II.), then to 44°C (III.) in our patient (left panel). The blue trace shows the perfusion measured in arbitrary units by the equipment (PU: perfusion unit), the red trace shows the temperature of the probe, and the red arrows on the top indicate the time points of heating. For comparison, the right panel shows the heat-induced perfusion increase of a healthy child. Note that the PU scale is set differently due to the severely reduced perfusion of the patient.

Periflux 5000 system (Perimed AB, Stockholm, Sweden) uses the Laser Doppler Flowmetry (LDF) technology for perfusion measurement. A thermostatic Laser Doppler probe was placed on the distal phalanx of the second finger of the right hand to measure the effect of local heating on skin microcirculation (42°C for 15 minutes then to 44°C for 5 minutes). Severely reduced basal blood flow was detected on the affected fingers with delayed vasodilatation after local heating (Table 2, Figure 2).

**Table 2:** Numerical data characterizing the baseline blood flow and the heat-induced perfusion increase in our patient and a healthy child, calculated from the measurements with the Periflux 5000 system*

	Mean perfusion (arbitrary units)		Latency to reach maximum (minutes)
	at 32°C	at 42°C	
Patient	36.14	126.74	4:12
Healthy	455.70	811.65	0:51

*Data from the 44°C stimulation not shown as we did not measure any apparent increase during this period compared with the 42°C heating.

**Figure 3:**
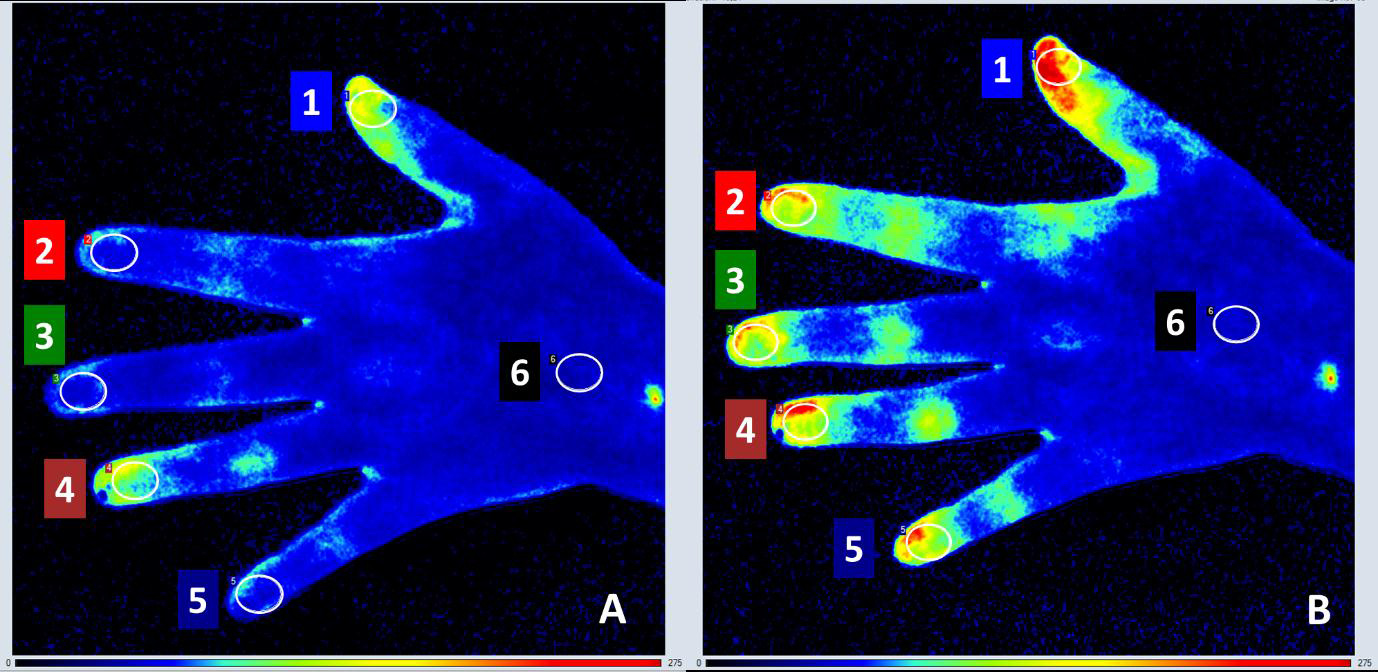
Representative images of Laser Speckle Contrast Analysis (LASCA) measurements. **A.** LASCA measurements performed on the left hand before pentoxyphyline infusion (200 mg IV) with the PeriCam PSI System. **B.** LASCA measurements performed on the left hand 1 hour after intravenous pentoxyphyline infusion (200 mg) with the PeriCam PSI System. The perfusion is measured in arbitrary perfusion units (PU) by the equipment and imaged on a color scale. Higher numbers and yellow-red color shades represent higher perfusion values. Equal-sized regions of interests (ROIs) were selected on each finger and on the dorsal side of the hand. The ROI outlines were enhanced and enlarged number legends for ROIs were added during image post-processing to allow visibility for publication.

The Laser Speckle Contrast Analysis (LASCA) technology used by the PeriCam PSI equipment (Perimed AB, Stockholm, Sweden) allows real-time cutaneous perfusion imaging of larger body parts in several regions of interest (ROIs) simultaneously. This measurement also showed reduced perfusion in the acral regions and a significant perfusion increase in response to an acute 2-h-long pentoxyphyline infusion (200 mg) 1 hour after finishing the treatment (Table 3, Figure 3).

**Table 3:** Mean perfusion values of entire regions of interest (ROI) of equal size representing each fingertip before and 1 hour after intravenous pentoxyphyline treatment (200 mg) with the PeriCam PSI System

	Mean perfusion (arbitrary units)		
ROI	before pentoxyphyline	after pentoxyphyline	Increase (%)
1st finger	122.80	212.39	73.0
2nd finger	67.49	135.78	101.2
3rd finger	62.73	132.73	111.6
4th finger	104.19	149.49	43.5
5th finger	69.96	139.38	99.2
Dorsum of the hand	49.68	56.30	13.3

At the follow-up visit after 6 months, the patient had mild symptoms, and the laboratory parameters returned to reference ranges. So far, she has not received any treatment for one and a half years or had any symptoms of vascular disease (Table 4).

**Table 4:** Timeline of patient’ signs and symptoms and treatment

Date	Signs and symptoms
November 10, 2014	Subfebrility, cough - amoxicillin-clavulanate antibiotic and fluticasone propionate, salbutamol inhalation treatments
November 17, 2014	Painful, bluish discoloration of fingers
November 24, 2014 admission to hospital	Severe acrocyanosis with cutaneous necrosis of the digital phalanx of the fourth finger, pneumonia l.d. – clarithromycin and pentoxyphyline treatment
second week of hospitalization	Systemic autoimmune diseases were ruled out, serological test suggested recent *Mycoplasma pneumoniae* infection
January 2015	LDF and LASCA methods
May 2015	The patient had mild symptoms, the laboratory parameters returned to reference ranges
February 2016	Last pentoxyphyline infusion
March 2016	No symptoms of vascular disease

## DISCUSSION

This is the first report describing a pediatric case of cold agglutinin-induced acrocyanosis after Mycoplasma infection without any other severe symptoms and laboratory alterations. The diagnosis of severe forms of acrocyanosis is generally challenging and it can take weeks to identify the background of the diagnosis. Therefore, we used LDF and LASCA techniques as non-invasive investigational tools to assess and quantify the severity of the perfusion disturbances and monitor the effectiveness of the treatment. However, there was no long-term follow-up of the microcirculatory changes and vascular responsiveness after complete remission of the symptoms.

Severe acrocyanosis with cutaneous necrosis rarely occurs in children and it is mostly reported in association with systemic autoimmune disorders, such as systemic lupus erythematosus, systemic sclerosis, infection-induced cold agglutinin disease, and drug-induced acrocyanosis (1,2,6). We excluded the presence of autoimmune diseases on the basis of the laboratory tests. Psychomotor stimulants used for attention-deficit/hyperactivity disorder could also provoke acrocyanosis, but this patient was not treated with this type of drugs. The most likely cause of severe acrocyanosis was infection-induced cold agglutinin disease, which was supported by the respiratory infection two weeks earlier, the detectable cold-agglutinin at room-temperature, and the positive serological assay for *M. pneumoniae* infection.

Cold agglutinin disease accounts for about 10%-20% of all autoimmune hemolytic anemias. While idiopathic and secondary malignant disorders affect elderly people, the secondary post-infectious CAS is seen in adolescents, young adults and sometimes in children, especially in those with chickenpox (3,7-10). Despite the high incidence of infections that induce cold agglutinins, the titer and thermal amplitude of the antibodies are only rarely present in the pathologic range; therefore, these infections are seldom complicated with CAS. Symptomatic CAS mostly co-occurs with acute hemolysis in *M. pneumoniae* infections when the patient is recovering from the pneumonia, in parallel with peak titers of cold agglutinins. The antibody occurs 2 weeks after the onset of the primary infection, reaches peak titer quickly, and may persist for 3-4 months. Hemolysis is self-limited, lasting for 1 to 3 weeks (4,7,11,12). However, in our patient, CAS manifested without hemolysis. Acrocyanosis of the fingers, toes, nose, and ears is caused by erythrocyte sludging in the microvasculature, but ulceration and necrosis are uncommon. Other findings are variable and depend on the underlying disease (3,4,13,14). Less commonly, other viral or bacterial pathogens, such as cytomegalovirus, influenza virus, varicella virus, Legionella, Citrobacter, and some strains of *Listeria monocytogenes* can also induce oligoclonal cold agglutinin production (8-11,15).

The disease variability depends on several pathophysiologic factors (16). IgM weakly binds to the polysaccharide antigen at ambient temperature, but the higher the thermal amplitude of the antibodies, the greater the possibility of reaching the critical temperature at the periphery. Although the thermal range is the most important factor, the clinical manifestation of cold agglutinins in these infections is invariably associated with elevated titers ranging between 512 and 32 000 (3). Another factor is the capability of the IgM to fix complement. Since phagocytic cells do not have IgM receptors, red blood cell destruction is complement-mediated. Some cold agglutinins do not fix complement and patients with such antibodies may have severe agglutination symptoms (acrocyanosis) without hemolysis (17-20).

We demonstrated the severely impaired perfusion of the patient’s fingers with two non-invasive techniques, the LDF and LASCA. Both microcirculation measurement techniques rely on the analysis of reflected light from moving red blood cells and the calculated perfusion unit is proportional to the number and velocity of circulating red blood cells in the examined tissue. While LDF is often used for the diagnosis and follow-up of peripheral vascular diseases, such as diabetic microangiopathy, atherosclerosis, wound healing after burns, or reconstructive surgery in adults (21-24), LASCA is a novel technique for the study of microcirculation without any routine applications in the clinics (25-27). Neither of the methods has been used in the pediatric practice. In the present case, we demonstrated the usefulness of both methods. Impaired perfusion was reliably detected by both LDF and LASCA. In addition, it was revealed that the heat-induced hyperemic response was diminished and improved microcirculation of the acral regions after pentoxyphyline infusion was sensitively measured by the LASCA imaging method.

The primary take-away lesson in this case is that if a child presents with atypical Raynaud’s-like symptoms, it should alert the clinician to suspect cold agglutinin disease. Cold agglutinin-induced acrocyanosis after Mycoplasma infection can develop in children without hemolysis.
